# Probable novel *PSEN2* Val214Leu mutation in Alzheimer’s disease supported by structural prediction

**DOI:** 10.1186/1471-2377-14-105

**Published:** 2014-05-15

**Authors:** Young Chul Youn, Eva Bagyinszky, HyeRyoun Kim, Byung-Ok Choi, Seong Soo An, SangYun Kim

**Affiliations:** 1 Department of Neurology, Chung-Ang University College of Medicine, Seoul, South Korea; 2 Department of Bionano Technology, Gachon Bionano Research Institute, Gachon University, Seongnam-si, South Korea; 3 Department of Laboratory Medicine, Chung-Ang University College of Medicine, Seoul, South Korea; 4 Department of Neurology, Samsung Medical Centre, Sungkyunkwan University, Seoul, South Korea; 5 Department of Neurology, Seoul National University Bundang Hospital, Seoul National University College of Medicine, Seongnam, South Korea

**Keywords:** Alzheimer’s disease, Presenilin 2 mutation, Presenilin 2 protein structure, Novel mutation, Structural prediction

## Abstract

**Background:**

*PSEN2* mutations are rare variants, and fewer than 30 different *PSEN2* mutations have been found. So far, it has not been reported in Asia.

**Case presentation:**

*PSEN2* mutation at codon 214 for predicting a valine to leucine substitution was found in a 70-year-old woman, who showed a dementia of the Alzheimer type. We did not find the mutation in 614 control chromosomes. We also predicted the structures of presenilin 2 protein with native Val 214 residue and Leu 214 mutation, which revealed significant structural changes in the region.

**Conclusion:**

It could be a novel mutation verified with structural prediction in a patient with Alzheimer’s disease.

## Background

Well-known mutations associated with autosomal dominant Alzheimer’s disease (AD) inheritance are amyloid precursor protein (*APP*) and presenilin 1 (*PSEN1*) and presenilin 2 (*PSEN2*) [1,2]. Previous studies have shown several *APP* and *PSEN1* mutations in Asian populations, but pathogenic *PSEN2* mutation has not yet been described [3,4]. We had screened *PSEN2* mutation in 90 AD patients of two memory clinics from May to December 2012. Here, a patient of East Asian descent with AD was found with a probable novel Val214Leu mutation at *PSEN2* exon 7.

## Case presentation

**Figure 1:**
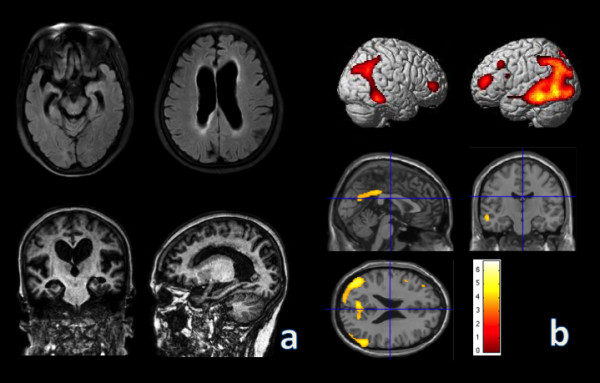
**Axial FLAIR, coronal and sagittal T1 images of brain MRI (a) and statistical parametric maps (21 age matched control subjects (70.3 ± 1.4 yr); p < 0.001, uncorrected; extent threshold = 10 voxels) of FDG-PET (b) of the patient.** The color scale of statistical parametric maps indicates the Z value magnitude, with the lowest value appearing in dark red and the highest value in bright yellow/white.

A seventy-year-old right-handed female has complained progressive memory problems, which started one year prior to the initial visit. She reported her frequent forgetfulness of going to important meetings and had difficulties in grocery shopping. She also reported in having a tremor in her right hand for the past 20 years, persistent during resting and forearm stretching stances, which did not progress further. She presented with slightly decreased facial expressions, but did not show rigidity, ataxia, myoclonus, or seizure. Since she was separated from her other family members during the Korean War, we could not obtain her family history. Her Korean version of Mini-Mental Status Examination (K-MMSE) was 18 and CDR-sum of boxes was 4.5. She also underwent neuropsychological tests using a standardized neuropsychological battery called the Seoul Neuropsychological Screening Battery (SNSB) [5]. She had normal spontaneous speech and comprehension. Her SNSB showed digit span forward was 16.86%ile; Korean-Boston naming test, 12.09%ile; Rey-Osterrieth Complex Figure Test copy, 0.01%ile; calculation, < 5%ile; Seoul Verbal Learning Test-delayed recall, 1.05%ile; Rey-Osterrieth Complex Figure-delayed recall test, 2.09%ile; Digit Symbol Coding, 1.15%ile; Korean Stroop test-color reading, 11.46%ile; Controlled Oral Word Association Test, 0.01%ile. She also revealed the BPO (body part as object) error when doing praxis test. Fist-edge-palm and alternating hand movement, motor impersistence test were normal, but Luria loop and alternating square and triangle were deformed and perseverative. Neuropsychological tests revealed severe verbal memory impairment, which was not improved by cues. In addition, she showed visuo-spatial dysfunction and difficulty in calculation and frontal executive function. Her brain MRI (Figure 1a) showed diffuse cortical atrophy with mild white matter hyperintensities in FLAIR images, and FDG-PET (Figure 1b) revealed bilateral temporoparietal and precuneus hypometabolism. A diagnosis of probable AD was made. She had an *APOE ϵ3/4* polymorphism, but other laboratory tests were normal.

## Genetic analysis of *PSEN2* and structural prediction of mutant PSEN 2 protein

DNA was extracted from the buffy coat of the venous blood samples using a blood DNA isolation kit (GeneAll Inc., Seoul, South Korea). *APP* exon 16 and 17, *PSEN 1* exon 4, 5, 6, 7, 8, and 11, *PSEN 2* exon 4, 5, 6, 7, and 12, the coding region of the *PRNP* gene, mutations in the microtubule-associated protein tau (*MAPT*) and progranulin (*GRN*) were amplified by PCR for genetic analysis. A sizing-genotyping and repeat-primed PCR were performed to monitor the presence of the abnormal repeat expansions of C9orf72.

**Figure 2:**
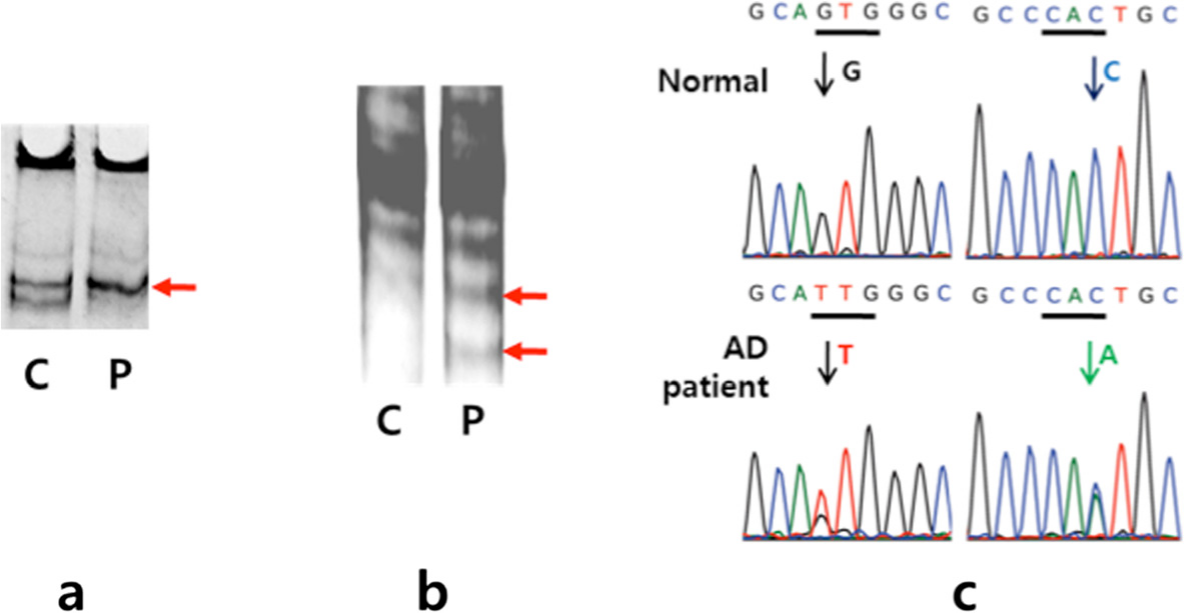
**Genetic analysis in finding novel Val214Leu mutation at *PSEN2 *exon 7.** After PCR amplification of *APP* exon 16 and 17, *PSEN 1* exon 4, 5, 6, 7, 8, and 11, *PSEN 2* exon 4, 5, 6, 7, and 12, and the coding region of the *PRNP* gene, PCR amplicons were analysis by single-strand conformation polymorphism (SSCP, **a**) and Surveyor nuclease assay **(b)**. PCR amplicon from AD patient showed different cleavage pattern than the normal control by nuclease, as indicated with red arrows **(c)**. All PCR amplicons were sequenced with primers, used in PCR amplification. Novel mutation of Val214Leu at *PSEN 2*at exon 7 of the patient was obtained, where GTG of Val was converted into TTG of Leu at 5′ direction (red arrow) and CAC to CAA at 3′ direction (green arrow).

Single-strand conformation polymorphism (SSCP) [6] and Surveyor nuclease assay kit [7] revealed alternative migration and cleavage patterns in comparison to normal (Figure 2a, b), strongly suggesting the presence of mutation in the PCR product of *PSEN 2* exon 7. All PCR products were sequenced for the confirmation. We found a valine to leucine substitution (Val214Leu) at exon 7 of *PSEN2* in both patients (Figure 2c). This *PSEN2* mutation could be considered as a novel mutation, since 614 control chromosomes (307 subjects: 140 healthy control, 90 AD, 15 mild cognitive impairment, 12 subjective memory impairment, 10 frontotemporal dementia, 5 vascular cognitive impairment, 35 unclassified patients) were also screened for Val214Leu mutation without any evidence, in support of the present finding. All study subjects provided written informed consent to allow their genetic and clinical data to be used for research purposes. The Institutional Review Board at Chung-Ang University Hospital and Seoul National University Bundang Hospital approved this study.

**Figure 3:**
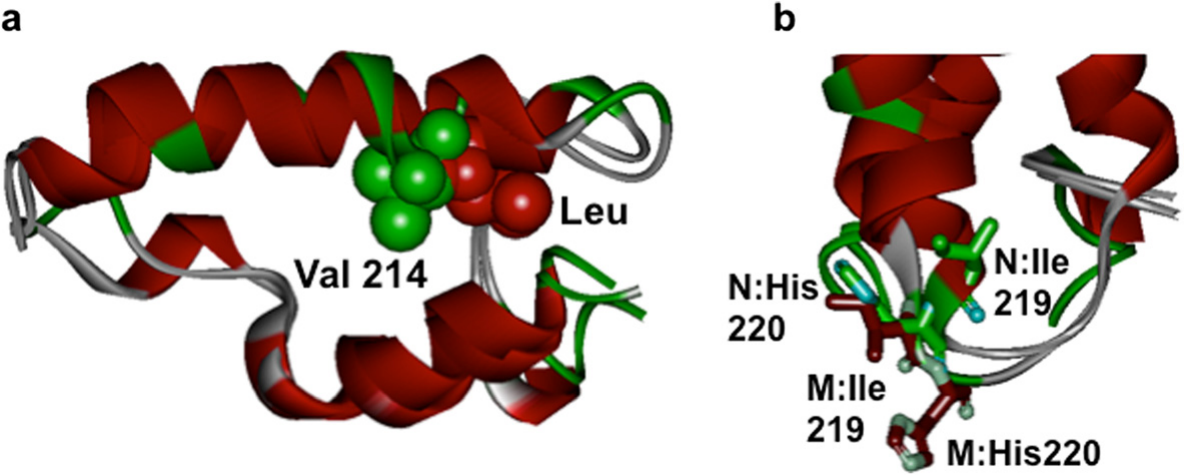
**Superimposed structures of presenilin 2 with native Val 214 residue and Leu mutation.** The structures of presenilin 2 with native Val 214 residue and Leu mutation were generated by RaptorX 3D prediction program. The side-chains of Val and Leu 214 residues reveal noticeable changes of extending towards opposite direction **(a)**. Green color CPK (Corey, Pauling, Koltun) indicates the side-chain of native Val residue and the red color indicated the Leu mutation. Other proceeding residues, Ile 219 and His220, revealed significant changes in their side-chains **(b)**. Green colored side-chains indicated the Ile and His residues from the native form and red colored side-chains indicated the mutant presenilin 2.

The structures of presenilin 2 with native Val 214 residue and Leu 214 mutation were generated by RaptorX 3D prediction program. Overall structural features were identical between the native presenilin 2 with Val 214 residue and Leu mutation. However, the side-chains of Val and Leu 214 residues reveal noticeable changes of extending towards opposite direction. In addition, other proceeding residues, Ile 219 and His220, revealed significant changes in their side-chains (Figure 3).

Polymorphism Phenotyping v2 (PolyPhen 2, http://genetics.bwh.harvard.edu/pph2/) online program for predicting the putative pathogenic phenotype was also used to evaluate *PSEN2* Val214Leu mutation by analyzing the non-synonymous mutations in the coding region [8]. PolyPhen2 compared the wild-type sequences and the mutant sequences by using sequence-and structure-based predictive features, based on maping the missense mutations to the transcriptomes, performes protein sequence annotations and structural comparisons. Next, PolyPhen 2 performed a multiple alignment of sequences from different organisms. In addition, it could provide 3D structure of the protein by searching protein data bank database. Two types of datasets, HumDiv and HumVar, were available from PolyPhen 2, which were calculated from position-specific independent counts (PSIC) scores of normal and mutant allele. For PSEN2 Val214Leu, the HumDiv score was 0.972 (sensitiviy: 0.77; specificity: 0.96), which indicated as probably damaging mutation with the HumVar score of 0.836 (sensitiviy: 0.73; specificity: 0.88), as possibly damaging mutation.

## Discussion and conclusion

We detected a single nucleotide polymorphism on exon 7 of *PSEN2* in a patient with dementia of the Alzheimer type. So far, a pathological *PSEN2* mutation has not been reported in Asia. Particularly, Val214Leu at *PSEN2* was not reported previously and also was not found in general Korean population. This is suggesting as a probable novel *PSEN2* mutation in association with this AD patient. Upon structural prediction of native and mutant presenilin 2 by using RaptorX program, the results revealed significant structural changes in the region (Figure 3). When two structures of Val 214 and Leu mutant were superimposed, the where side-chains of Val 214 and Leu 214 were pointing to opposite direction, suggesting drastic alterations in the region. Additional residues, Ile219 and His220, also revealed dramatic changes, supporting that Val replacement to Leu could cause overall structures and function of presenilin 2 (unpublished result, An’s personal communication). On the other hand, many other pathogenic mutations of Val replacement to Leu were reported [9,10].

PolyPhen2 and other bioinformatic algorithms could be used for predicting the pathogenic nature of novel mutations or known mutations [11,12]. Since cell models and in *vitro/in vivo* experiments would be costy, *in silico* estimations and simulations could aid in understanding the pathogenic or non-pathogenic nature of mutations. The prediction for *PSEN2* Val214Leu by Polyphen2 provided HumDiv and HumVars scores of 0.972 and 0.836. Hence, These analyses suggested the *PSEN2* Val214Leu mutation could be a possibly strong damaging variant.

Multiple sequence alignment for *PSEN2* was performed in comparison with different organisms. Analysis predicted that the vertebrates, such as rhesus macaque, african elephants pigs, dog, duck, revealed the normally codon Val at 214. However, few organisms (invertebrates, mostly unicellular organisms) revealed the alterations, Leu, Gly, Ile or Met, at the same Val214 position. For example, Methionine, isoleucene, or leucine were found in the *Tribolium castaneum*, *Trichoplax adhaerens*, and *Entamoeba histolytica*, respectively. *PSEN2* V214 seemed to be strongly conserved in 115 species and mutations of Leu, Gly, Ile or Met were found in 8 species.

AD patients who have exon-7 *PSEN2* have reported wide spectrum of cognitive and non-cognitive symptoms. They could initially show anxiety and irritability along with mild deficits in memory, attention, and language. Then they could also show seizure and myoclonus. Some patients present global cognitive impairment at first. Exon-7 *PSEN2* mutation revealed an age range of onset between 45 and 85 years overlapping early and late onset AD [13-15]. This could be explained by the demonstrated tendencies of *PSEN2* mutations in presenting lower penetrance and appearing a later and more variable age of onset than the other early-onset AD gene mutations [16]. Although the patient showed a multi-domain cognitive impairment with decreased facial expression and non-progressing right hand tremor, her significant memory impairment was the initial main complaint at age 69 and prominent temporoparietal lobe dysfunction supported by FDG-PET, no abnormal repeat expansion of C9orf72 and no other mutation in MAPT and GRN, which could be clinically classified as late-onset AD. Because her family was separated during Korean War, family history was limited. We could not find any other affected family member.

When considering diagnoses atypical AD patients, we can do CSF Aβ 1-42 analysis or Pib-PET supporting an AD diagnosis. Even though she did not perform CSF and Pib-PET study, we considered the patient as probable AD because she initially complained memory problem confirmed with a neuropsychological test and showed bilateral hippocampal atrophies in structural MRI and hypometabolism of bilateral temporoparietal area and precuneus in FDG-PET.

Even though, this report is providing limited evidence of pathogenicity, to our knowledge, this is the first case report of AD with this probable novel *PSEN2* Val214Leu mutation verified with structure prediction.

### Consent

Written informed consent was obtained from the patient for publication of this Case report and any accompanying images. A copy of the written consent is available for review by the Editor of this journal.

## Competing interests

The authors declare that they have no competing of interests.

## Authors’ contributions

YCY: drafting/revising the manuscript for content, analysis and interpretation of data. EB and SSAn: performing the genetic analyses, predicting presenilin 2 protein structure and interpretation of the data. HRK: preparing the samples and drafting/revising the manuscript for content. BOC: verifying the mutations in the general population, and interpretation of data. SYK: analysis or interpretation of data, study supervision, obtaining funding. All authors read and approved the final manuscript.

## Authors’ information

This is a case report. This manuscript has not been published elsewhere, nor is it under simultaneous consideration for publication elsewhere. All of the authors and contributors have agreed to the conditions of authorship. All authors have full access to all of the data and have the right to publish any and all data, and each author believes that the manuscript represents honest work. The methods section includes a statement of our institutional review board (IRB)’s approval. This research complies with the principles of the Declaration of Helsinki (1964). No identifying information for the patient is included in the data reported. There are no conflicts of interest to report and all authors adhered to ethical standards.

## Pre-publication history

The pre-publication history for this paper can be accessed here:

http://www.biomedcentral.com/1471-2377/14/105/prepub
